# Survey design to assess condition of wetlands in the United States

**DOI:** 10.1007/s10661-019-7322-6

**Published:** 2019-06-20

**Authors:** Anthony R. Olsen, Thomas M. Kincaid, Mary E. Kentula, Marc H. Weber

**Affiliations:** U.S. Environmental Protection Agency, ORD/National Health and Environmental Effects Laboratory, Western Ecology Division, 200 S.W. 35th Street, Corvallis, OR 97333-4902 USA

**Keywords:** Wetlands, Probability survey design, Wetland condition, Frame imperfections, Non-response

## Abstract

The US Environmental Protection Agency (US EPA) initiated planning in 2007 and conducted field work in 2011 for the first National Wetland Condition Assessment (NWCA) as part of the National Aquatic Resource Surveys (NARS). It complements the US Fish and Wildlife Service (USFWS) National Wetland Status and Trends (S&T) program that estimates wetland acres nationally. The NWCA used a stratified, unequal probability survey design based on wetland information from S&T plots to select 900 sites for the conterminous 48 states. Based on site evaluation information, the NWCA estimates that there are 94.9 (± 6.20) million acres of wetlands in the NWCA target wetland population (reported in acres to be consistent with S&T). Not all of the estimated target population acres could be sampled due to accessibility and field issues. Based on the sites that could be sampled, the sampled population for the NWCA is estimated to be 62.2 (± 5.28) million acres of wetland area. Landowner denial for access was the main reason (24.7% ± 3.5%) for the sampled population being smaller than the target population, and physical inaccessibility was the second reason (6.8% ± 2.1%). The NWCA 2011 survey design was successful in enabling a national survey for wetland condition to be conducted and coordinated with the USFWS S&T survey of wetland extent. The NWCA 2016 survey design has been modified to address sample frame issues resulting from the difference in S&T focusing only on national estimates and NWCA focusing on national and regional estimates.

## Introduction

In 2007, the US Environmental Protection Agency (US EPA) initiated planning for the National Wetland Condition Assessment (NWCA) as part of the National Aquatic Resource Surveys (NARS). The NWCA conducted field work in 2011 and was the fourth aquatic resource surveyed by NARS. It was preceded by coastal water, lake and reservoir, and river and stream assessments conducted in 2007 to 2010. The NWCA is the first national assessment of wetland condition conducted within the USA and complements the US Fish and Wildlife Service (USFWS) National Wetland Status and Trends (S&T) program, which has provided national estimates on wetland area over time since its inception in 1974 (Frayer et al. 1983; Dahl et al. 1991; Dahl 1990, 2000, 2006, 2011; Dahl and Bergeson 2009).

In the early 1990s, the US EPA’s Environmental Monitoring and Assessment Program (EMAP) conducted research on approaches to monitoring wetland condition (Leibowitz et al. 1991). Their goal was “to provide a quantitative assessment of the current status and long-term trends in wetland condition on regional and national scales.” Leibowitz et al. (1993) considered alternative survey designs and conducted pilot studies to investigate the design’s feasibility (Ernst et al. 1995; Turner et al. 1995; Lesser 2001). The NWCA is based on that research. Wardrop et al. (2007a, b), Stevens Jr. and Jensen (2007), Whigham et al. (2007), Jacobs et al. (2010), and Fennessy et al. (2007) describe assessments of wetlands in the Juniata watershed in Pennsylvania, the Nanticoke watershed in Maryland and Delaware, and the Cuyahoga watershed in Ohio. At a larger scale, Nestlerode et al. (2009, 2014) report on an assessment of Gulf of Mexico coastal wetlands. Wardrop et al. (2013) describe the evolution of monitoring and assessment in the Mid-Atlantic, culminating in an assessment of wetlands in the region. These studies used unequal probability survey designs, investigated potential indicators of wetland condition, and demonstrated the technical capability for conducting wetland assessments at the scale of a watershed and larger. At the same time, state wetland programs were also testing and implementing wetland assessment efforts. The State of Minnesota is particularly noteworthy because it has employed a probability design to assess the quantity and quality of its wetlands (Genet and Olsen 2008, Genet 2012). This work was augmented by a state-wide assessment of wetland condition implemented as part of the 2011 NWCA and used a two-phase probability design (Minnesota Pollution Control Agency 2015).

This paper describes the 2011 NWCA probability survey design, including its implementation and statistical estimation procedures. Since the geographic information system spatial coverage of wetland polygons (i.e., the sample frame) may not always correctly identify the wetlands included in the NWCA, the paper provides estimates of the proportion of the sample frame wetland area that is included in the NWCA. Finally, all wetlands included in the NWCA may not be able to be sampled either due to landowners denying field crews access to the wetland or due to the inaccessibility of the wetland site related to field crew safety or excessive time or cost required to do the assessment. The information from the evaluation status of the sites is used to estimate the proportion of the NWCA wetlands that could be sampled (i.e., the sampled population).

## Methods

### Study design

The wetland types included in the NWCA (i.e., the target population) include all tidal and non-tidal wetlands within the conterminous USA with rooted vegetation and when present, shallow open water less than 1 m in depth that is not currently being used in the production of crops. A wetland’s jurisdictional status under state or federal regulatory programs does not factor into the NWCA wetland-type definition. The NWCA uses a subset of the USFWS S&T wetland categories (Table 1), excluding marine intertidal (near shore coastal waters) and estuarine intertidal unconsolidated shore (beaches, bars, mudflats). Typically, intertidal categories occur in deeper water or are unlikely to contain rooted vegetation.

**Table 1:** General correspondence between US FWS S&T Wetland Category and NWCA target wetland type

S&T code	NWCA wetland type	Description of wetlands in NWCA wetland type
E2EM	EH	Estuarine intertidal (E) emergent (H = herbaceous)
E2SS	EW	Estuarine intertidal (E) forested and shrub (W = woody)
PEM	PRL-EM	Emergent wetlands (EM) in palustrine, shallow riverine, or shallow lacustrine littoral settings (PRL)
PSS	PRL-SS	Shrub-dominated wetlands (SS) in palustrine, shallow riverine, or shallow lacustrine littoral settings (PRL)
PFO	PRL-FO	Forested wetlands (FO) in palustrine, shallow riverine, or shallow lacustrine littoral settings (PRL)
Pf	PRL-f	Farmed wetlands (f) in palustrine, shallow riverine, or shallow lacustrine littoral settings (PRL); only the subset not currently in crop production
PUBPAB^a^	PRL-UBAB	Open-water ponds and aquatic bed wetlands (UBAB) in palustrine, shallow riverine, or shallow lacustrine littoral settings (PRL)

^a^PUBPAB is comprised of S&T Wetland Categories: PAB (palustrine aquatic bed), PUBn (palustrine unconsolidated bottom, natural characteristics), PUBa (aquaculture), PUBf (agriculture use), PUBi (industrial), and PUBu (PUB urban)

Since it is not feasible, or cost-effective, to sample all wetlands of interest, the NWCA approach to study design is to use a probability survey design. This enables estimates to be made for all wetlands of interest based on the results of the sample. Survey designs have some inherent characteristics that distinguish them from other sampling designs. First, the population being sampled (target population) is explicitly described. Second, every element in the population has the opportunity to be sampled with known probability that is greater than 0. Third, the selection process includes an explicit random element. In addition, a decision was made to have the NWCA study design linked to the USFWS S&T survey design, thus connecting the national reporting on status and trends in wetland condition with that on wetland area.

The NWCA wetland target population is viewed as a continuous resource; that is, wetlands are considered areal features. Any attribute of the wetland population, such as ecological condition, is assumed to vary continuously across the wetland. Consequently, the unit sampled is a site defined as a point where each attribute measured has a field plot design supporting its measurement. The plot design is described in detail in the NWCA 2011 Field Operations Manual (US EPA 2011).

### Sample frame

Preferably, a national geographic data layer that included polygons representing wetlands in the target population (i.e., the sample frame) would be available and used to select sample sites. When the survey design for NWCA was required, no such sample frame was available. The S&T survey design provides a sample of approximately 5000 2-mi × 2-mi plots from approximately 780,000, such plots covering the conterminous US. The USFWS S&T program delineated polygons in each plot in the sample based on 2005 aerial photography to identify S&T wetland types (Dahl and Bergeson 2009). This provides a nationally consistent set of wetland polygons based on current imagery.

The seven specific S&T wetland types used for the NWCA sample frame are E2EM, E2SS, PEM, PSS, PFO, Pf, and PUBPAB, where PUBPAB includes the PAB, PUBa, PUBf, PUBi, PUBn, and PUBu categories used by S&T (Table 1). These 171,834 wetland polygons from 4425 S&T plots (the remaining S&T plots did not include NWCA wetland type polygons) were then associated with attributes required for the NWCA survey design: state, EPA region, Omernik ecoregion level III (Omernik 1987; Omernik and Griffith 2014), and the level III ecoregions aggregated into three and nine ecoregions used by the NARS (Olsen and Peck 2008; Peck et al. 2013). The sample frame based on S&T plots was replaced for two states—Minnesota and Ohio. Ohio elected to base their survey design on a current digital map of wetlands in Ohio. Minnesota has a Wetland Status and Trends Monitoring Program (WSTMP) that assesses the status and trends of wetland quantity and quality in Minnesota (Kloiber 2010). Their wetland quantity survey is modeled after the USFWS S&T program. The WSTMP sample frame uses a grid that matches the USFWS S&T 4-mi^2^ grid boundaries. Each 4-mi^2^ grid cell was subdivided into four 1-mi^2^ grid cells. To be included in the design, at least 25% of a grid cell must be within the state. Similar to S&T, 4740 1-mi^2^ plots from the WSTMP sample frame were delineated using aerial imagery from 2006, 2007, and 2008 to create wetland polygons. These polygons constitute the NWCA sample frame for Minnesota. For each of the sample frames, the wetland-type codes used by the organization were mapped to match the NWCA wetland-type codes derived from USFWS S&T.

### Survey design

A complex survey design was required to meet the multiple objectives of the NWCA. Objectives included requirements to report on wetland condition nationally for seven wetland types, to report on nine NARS aggregated Omernik ecoregions, and to ensure that each of the states had a minimum of eight wetland sites to monitor. The random selection of the sites was completed in two steps (or phases) with the final inclusion probabilities being the product of the first step inclusion probability and the second step inclusion probability (which is conditional on the first step). The first step in the survey design combined wetland polygons from 46 states from the USFWS S&T plots, Minnesota WSTMP plots, and Ohio state-wide digital wetland map. In the next step, a Generalized Random Tessellation Stratified (GRTS) survey design for an area resource was applied to the wetland polygons (Stevens Jr. and Olsen 2004; Olsen et al. 2012). This step was stratified by state with unequal inclusion probabilities by the seven wetland-type categories within each stratum. Since guaranteeing the exact number of sites by wetland type was not important and classification of the wetland type in the sample frame was not perfect, unequal inclusion probabilities were used within a state. For the NWCA, the expected sample size was 900 sites for the conterminous 48 states, although states had the option of sampling additional sites. Allocation of sites by state and wetland type categories was completed by solving a quadratic programming problem that minimized the sum of the squared deviations of the expected sample size minus proportional allocation of sites by wetland type based on state area within each wetland type subject to constraints that (1) expected sample sizes by wetland type nationally was E2EM = 128, E2SS = 127, PEM = 129, PSS = 129, PFO = 129, Pf = 129, and PUBPAB = 129; (2) the minimum number of sites for a state was 8 (set to ensure all states participated in the NWCA); (3) the maximum number of sites within a coastal state for E2EM or E2SS was 13 to ensure that all coastal states were involved; (4) the maximum number of sites within a state for PEM, PSS, PFO, Pf, or PUBPAB was 10; and (5) the minimum number of sites was greater than or equal to zero for each wetland type and state combination. This approach ensured that the sample size for the seven wetland types was sufficient for national reporting and that each state received a minimum number of sites (which also improved the national spatial balance of the sites). It also ensured the proportional allocation the sites by area within a wetland type. Site selection was completed using the R package “spsurvey” (Kincaid and Olsen 2015).

The total number of site visits was 996 allocated to 900 unique sites with 96 sites to be revisited (two per state). To ensure that a sufficient number of sites were available that could be sampled, an additional 900 sites, as an over-sample, were selected to provide replacement sites for those sites that either are not part of the target population or could not be sampled (permission to sample not given by the landowner or site was not able to be sampled due to other access issues). To ensure that the final set of sites evaluated satisfied the requirements for a probability survey design, the sites were ordered in reverse hierarchical order (Stevens Jr. and Olsen 2004), and all sites from the first site in the list until the last evaluated site in the list were included in the study.

Three states elected to modify the survey design for their state. The state modifications replaced the above survey design for their state. In each case, the state designs identified sites that were required for the NWCA and additional sites that were specific to the state to meet state requirements. Wisconsin elected to study the Southeastern Plains Till region (Omernik and Griffith 2014). This was accomplished by the USFWS S&T team selecting additional 4-mi^2^ plots within the study region. For the NWCA survey, the Wisconsin state stratum was replaced by a new design that included two strata, the Southeastern Plains Till region and the rest of the state. The sites selected under the national NWCA design were used for the rest of Wisconsin state region, and a new GRTS unequal probability survey design of 50 sites was selected for the Southeastern Plains Till region. Unequal inclusion probability categories were the five S&T wetland types PEM, PSS, PFO, Pf, and PUBPAB.

Ohio elected to base their survey design on a current digital map of wetlands in Ohio (http://www.ducks.org/conservation/glaro/glaro-gis-nwi-update-data#oh). A sample of size 50 was selected using an areal GRTS unequal probability survey design. The unequal probability categories were S&T wetland types PEM, PSS, PFO, Pf, and PUBPAB.

Minnesota elected to base their survey design on mapped wetlands in the 1-mi^2^ WSTMP plots. The next step was to select 150 sample sites using a GRTS unequal probability survey design from the delineated wetland polygons. The Minnesota sites required for the NWCA were the first 22 sites that were sampled when ordered by their site identification. An additional 150 sites were selected for use if any of the initial 150 sites could not be sampled, using the same process described earlier.

### Wetland evaluation and field sampling

A critical element in the implementation of the survey design is the determination of the status of each site in the sample relative to the requirements of the design. Each site was checked to determine if it met the target population definition of a wetland included in the study. Where possible, the determination was made without a site visit; however, field reconnaissance was necessary for some evaluations. Initially, sites were screened using aerial photo interpretations to identify locations that did not meet the target population definition (e.g., non-NWCA wetland types, wetlands converted to non-wetland land cover due to development). Two other situations resulted in a wetland site not being sampled. First, many wetlands are on private land and require landowner permission to access. All landowner refusals were documented and recorded. Secondly, some wetlands are physically inaccessible, e.g., would require helicopter to access or 2–3 days to reach by canoe. When logistical or safety constraints made a wetland site inaccessible, the reason was recorded.

### Calculating the sample weights

A critical activity for analyzing data from a study with a stratified, unequal probability survey design is deriving the weights for each of the evaluated sites. For this study, wetland area was used to determine the inclusion probability for each site. All statistical analyses used the weights based on the inclusion probability for each site, i.e., the inverse of its inclusion probability. Initial weights were calculated for each site based on the unequal inclusion probabilities used to select the sites based on the design. Since the site selection involved two steps, the initial weights are the product of the weights from the first step of selecting 4-mi^2^ plots and the weights from the second step of selecting sites from wetland polygons identified in the selected 4-mi^2^ plots. The final weights for the first step are based on the USFWS S&T strata (combination of state and physiographic region) modified to account for designs in Minnesota, Wisconsin, and Ohio. Final weights are equal to the total stratum area divided by the total area of the plots selected. This is an approximation of the number of 4-mi^2^ plots in the stratum divided by the number of 4-mi^2^ plots selected in the stratum. This is required since the plots are not all 4-mi^2^ plots due to state boundaries. Weights for the second step are then adjusted by state and are equal to the initial weight for a site multiplied by the ratio of the total wetland acres in the 4-mi^2^ plots within the state divided by the sum of the initial weights for all sites evaluated within the state. This adjustment accounts for the use of additional sites evaluated when the initial sites could not be sampled for various reasons and the requirement that each state sampled the number of sites specified by the design.

### Population estimation

Information from the wetland site evaluation process was used to estimate the number of acres of wetlands in the target population for NWCA. The site evaluation information from sites determined to be target wetlands (whether sampled or not) was used to estimate the acres of wetlands that would not be sampleable (landowner denial or physically inaccessible) if they were selected. Finally, the site evaluation information from sites determined to be non-target for NWCA was used to investigate deficiencies of the sample frame. Diaz-Ramos et al. (1996) describe the statistical procedure used to produce these estimates. For calculating margins of error for these estimates, we used a variance estimate called a local neighborhood variance estimate, appropriate for spatially balanced survey designs, developed by Stevens Jr. and Olsen (2003). Analyses were completed using the R statistical software (R Core Team 2015) and the R contributed package *spsurvey* (Kincaid and Olsen 2015) for probability survey population estimation. In most cases, the local neighborhood variance estimator yields confidence interval coverage closer to the expected coverage than the standard Horvitz-Thompson variance estimator, which is typically used in complex, variable-probability survey designs (Stevens Jr. and Olsen 2003). The estimated proportion (*p*_c_) in an evaluation category is as follows:

$$ {p}_{\mathrm{c}}=\frac{\sum_{\mathrm{i}=1}^{\mathrm{n}}{w}_{\mathrm{i}}\ast {x}_{\mathrm{i}}}{\sum_{\mathrm{i}=1}^{\mathrm{n}}{w}_{\mathrm{i}}} $$

where *x*_i_ = 1 if the site evaluation category *c* occurs for the *i*th wetland site and equals 0 otherwise, *w*_i_ = the adjusted weight for *i*th wetland site, and *n* = total number of wetland sites evaluated.

## Results

**Fig. 1:**
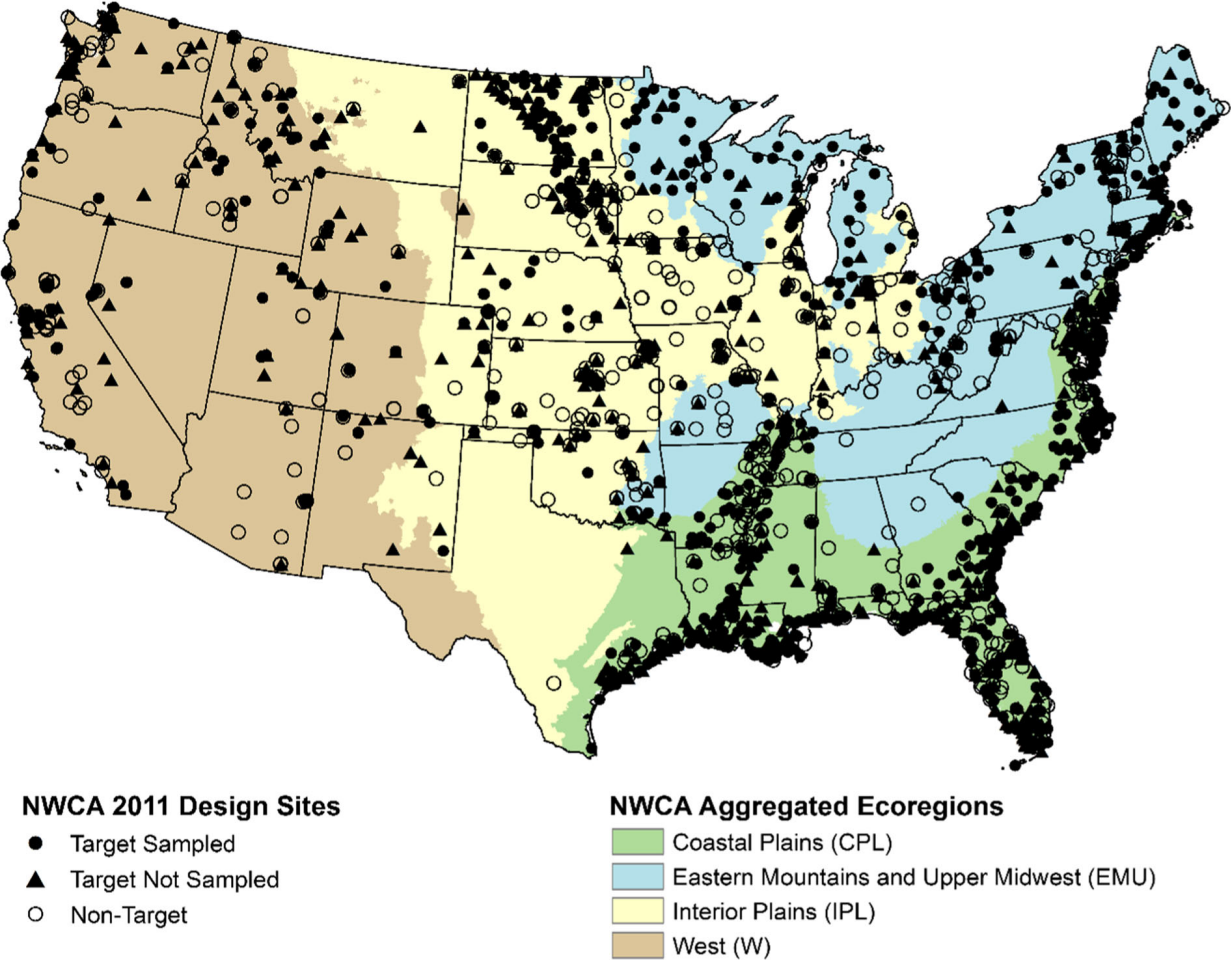
Wetland sites evaluated in NWCA 2011 (*n* = 2313) identified by those sampled, those evaluated to be target but could not be sampled, and those evaluated to be non-target

To sample 967 sites in the field, 2313 sites were evaluated (Fig. 1) to determine whether the sites met the definition of a wetland for the NWCA 2011, and if it met the definition, whether the site could be sampled. The 967 sites included additional sites from Wisconsin and Ohio who used NWCA field protocols for their state surveys, so that their data could be used for NWCA 2011. The survey design was intended to report by three and nine aggregated ecoregions. Limitations in the number of sites available for the development of the vegetation index and availability of reference wetlands reduced the number of geographic regions and wetland classes for which wetland results could be reported. In the West, reduced sample size was also a factor limiting the number of reporting categories. Figure 1 shows four geographic regions used for NWCA reporting: Coastal Plains (CPL), Eastern Mountains and Upper Midwest (EMU), Interior Plains (IPL), and West (W). In addition, estuarine wetlands are reported only nationally (ALL). It was also necessary to collapse the original seven NWCA wetland types to four wetland types (Table 2) due to the small sample sizes within the reporting region, which limited the development of the vegetation indicator. The classes used were woody wetlands in palustrine, shallow riverine, or shallow lacustrine littoral settings (PRLW) combining PSS—shrub and PFO—forested; herbaceous wetlands in palustrine, shallow riverine, or shallow lacustrine littoral settings (PRLH) combining PEM—emergent, PUBPAB—open-water ponds and aquatic bed, and Pf—farmed wetlands not in current crop production; and the original estuarine wetland types of estuarine herbaceous wetlands consisting of E2EM wetlands (EH) and estuarine woody wetlands consisting of E2SS wetlands (EW).

**Table 2:** Definition of NWCA target population and description of seven NWCA wetland types and four aggregated wetland types

Target population		NWCA wetland type	Aggregated wetland type	Description
Wetlands across conterminous USA representing tidal and non-tidal systems that have rooted vegetation and, when present, open water ≤ 1-m deep	Estuarine (E)	EH: estuarine intertidal emergent	EH: estuarine intertidal herbaceous	Estuarine intertidal emergent
		EW: estuarine intertidal shrub/forest	EW: estuarine intertidal woody	Estuarine intertidal shrub and forested wetlands
	Inland (I)	PRL-EM: palustrine, riverine, and lacustrine emergent	PRLH: palustrine, riverine, and lacustrine herbaceous	Emergent, ponded, or previously farmed wetlands in palustrine, shallow riverine, or shallow lacustrine littoral settings
		PRL-UBAB -Palustrine, riverine, and lacustrine unconsolidated bottom/aquatic bed		
		PRL-f: palustrine, riverine, and lacustrine farmed (not actively farmed)		
		PRL-SS: palustrine, riverine, and lacustrine shrub/scrub	PRLW: palustrine, riverine, and lacustrine woody	Forest or shrub dominated wetlands in palustrine, shallow riverine, or shallow lacustrine littoral settings
		PRL-FO: palustrine, riverine, and lacustrine forested		

Of the sites evaluated, 1644 sites met the definition for an NWCA wetland and 669 did not (Table 3). The latter reflects the ability of the sample frame, which was based primarily on USFWS S&T wetland polygons to match the NWCA wetland target population definition. Landowner denial of access to sample a wetland site is the primary reason 1644 target sites had to be evaluated to obtain 967 sampled sites. Note that the number of sites in Table 3 cannot be used directly to interpret the quality of the sample frame and ability to sample NWCA wetland types. Rather, estimates based on the weights must be used to estimate the quality of the sample frame.

**Table 3:** Number of wetland sites evaluated by NWCA wetland type and evaluation status

Site evaluation	All	Wetland type						
		EH	EW	PRL-EM	PRL-UBAB	PRL-f	PRL-SS	PRL-FO
Target site								
Sampled	967	258	69	262	18	22	115	223
Access denied	429	22	48	135	57	37	67	63
Inaccessible effort	121	23	29	19	6	10	17	17
Inaccessible safety	5	2	–	–	–	–	3	–
AA_Area	14	–	3	1	5	1	2	2
AA_HGM	2	–	–	1	1	–	–	–
AA_Size	71	2	6	9	24	6	10	14
Proximity	11	–	3	–	1	7	–	–
Other	24	1	1	5	3	4	4	6
Total	1644	308	159	432	115	87	218	325
Non-target site								
Active crop	204	–	–	18	6	169	8	3
Aquaculture	100	1	–	5	83	8	1	2
Inundated	95	3	2	10	68	3	4	5
Not NWCA wetland	87	9	1	9	25	4	19	20
Not a wetland	183	3	2	45	43	41	24	25
Total	669	16	5	87	225	225	56	55
Total	2313	324	164	519	340	312	274	380

See Table 1 for definitions of wetland type acronyms. AA = assessment area; HGM = hydrogeomorphic; AA_Size = area too small (< 0.5 ha) to establish AA; AA_HGM = AA establishment would require crossing HGM boundaries; AA_Area = AA establishment would require inclusion of too much (> 10%) unsamplable area); proximity = AA would be too close to another NWCA sampling point (e.g., AA and/or buffer would overlap)

**Fig. 2:**
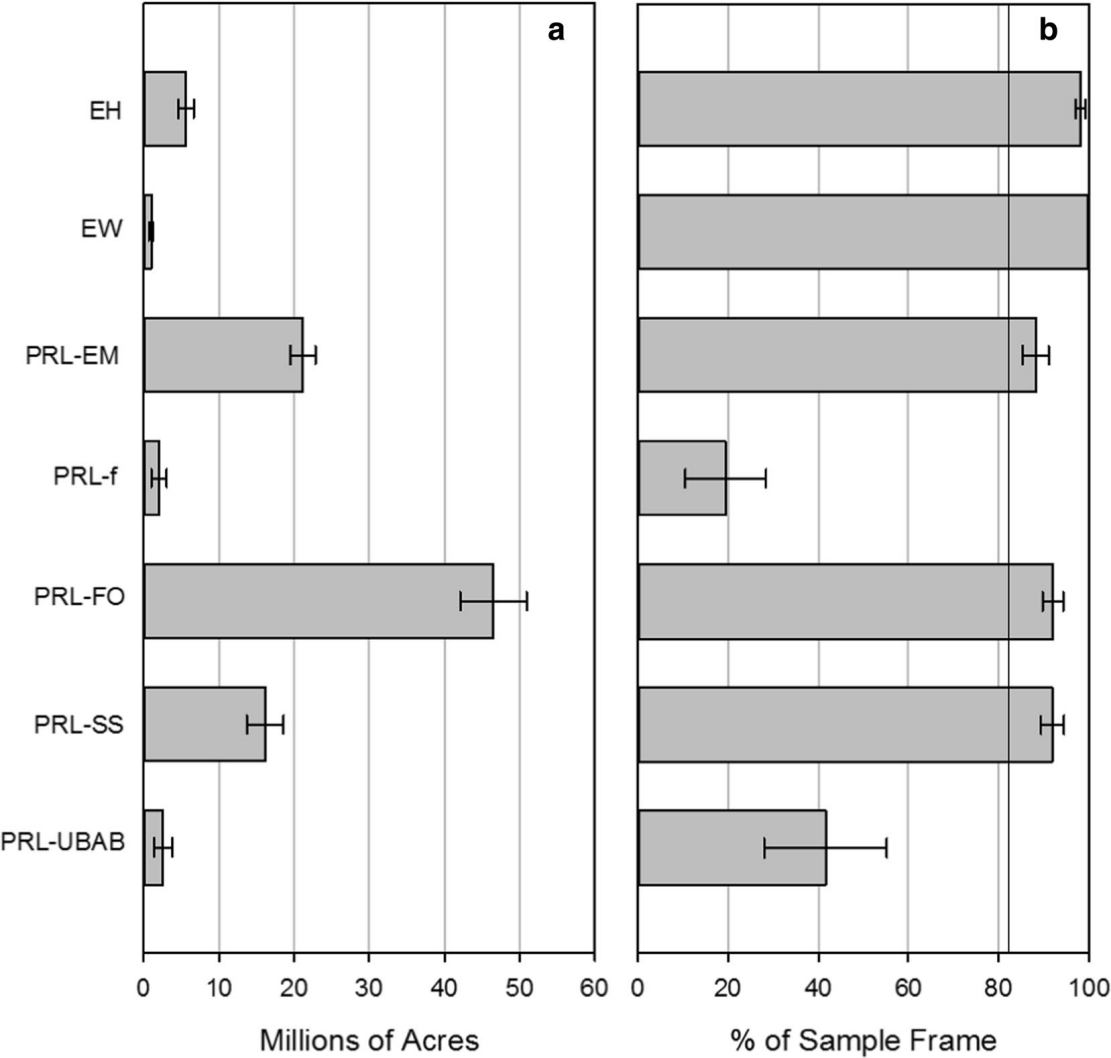
NWCA 2011 wetland type estimated area (millions of acres) for the target population (**a**) and as the percent of the sample frame area (**b**). See Table 1 for definitions of acronyms. The vertical line in panel **b** indicates the percent of the time the sample frame is correct for all wetland types. Error bars are 95% confidence intervals

Based on the site evaluation information, the NWCA estimated that there are 94.9 (± 6.20) million acres of wetlands in the NWCA target wetland population (Table 4). Of those, forested wetlands (FO) in palustrine, shallow riverine, or shallow lacustrine littoral settings (PRL) comprise 46.5 (± 4.39) million acres; emergent wetlands (EM) in PRL comprise 21.2 (± 1.65) million acres; shrub-dominated wetlands (SS) in PRL comprise 16.1 (± 2.43) million acres; estuarine (E) intertidal emergent (H = herbaceous) comprises 5.6 (± 1.04) million acres; open-water ponds and aquatic bed wetlands (UBAB) in PRL comprise 2.6 (± 1.22) million acres; farmed wetlands (f) in PRL comprise 2.0 (± 1.03) million acres; and estuarine (E) intertidal forested and shrub (W = woody) comprise 1.0 (± 0.30) million acres (Fig. 2a). With the exception of PRL-f (20%) and PRL-UBAB (42%), the sample frame wetland type correctly identified the NWCA wetland type over 88% of the time (Fig. 2b). For all NWCA wetland types, the sample frame wetland type was correct 82.4% (± 2.1) of the time, reflecting dominance in number of acres by PRL-FO, PRL-EM, and PRL-SS.

**Table 4:** Extent of wetlands nationally and by NWCA 2011 wetland type

Description	National (1000 acres)	National % (MoE)	EH % (MoE)	EW % (MoE)	PRL-EM % (MoE)	PRL-UBAB % (MoE)	PRL-f % (MoE)	PRL-SS % (MoE)	PRL-FO % (MoE)
Extent of wetlands in the US calculated using S&T Categories (1000 acres)	115,182 (± 6158) (46,613 ha)		5707 (± 1039)	991 (± 301)	23,986 (± 1650)	6172 (± 1416)	10,269 (± 1233)	17,550 (± 2399)	50,506 (± 4248)
Wetlands that were included in the S&T but did not meet criteria for “target” in NWCA for the following reasons:	20,268 (± 1860) (8202 ha)	17.6% (± 2.1%)	1.9% (± 1.1%)	0.2% (± 0.2%)	11.8% (± 2.9)	58.4% (± 13.5%)	80.5% (± 8.9%)	8.1% (± 2.5%)	8.0% (± 2.3%)
Wetland was actively cropped	9728 (± 1688) (3937 ha)	48.0% (± 6.3%)			24.7% (± 11.5%)	10.7% (± 12.3%)	91.6% (± 6.4%)	34.1% (± 12.6%)	14.6% (± 7.7%)
Wetland was not a wetland	3550 (± 784)	17.5% (± 3.7)	9.4% (± 10.9%)	5.9% (± 10.6%)	31.0% (± 12.0%)	14.6% (± 6.2%)	3.7% (± 2.8%)	27.8% (± 13.6%)	35.6% (± 12.6%)
Wetland type was not included in the NWCA target population	3186 (± 989) (1289 ha)	15.7% (± 4.9%)	59.3% (± 27.4%)	39.5% (± 56.5%)	15.0% (± 11.0)	8.7% (± 4.4%)	4.5% (± 6.1%)	30.6% (± 10.0%)	39.1% (± 11.4%)
Wetland was too deep (> 1 m)	2550 (± 883) (1032 ha)	12.6% (± 4.0%)	22.7% (± 21.1%)	54.7% (± 55.7%)	19.9% (± 14.6%)	43.4% (± 14.6%)	0.2% (± 0.2%)	7.4% (± 8.1%)	6.9% (± 6.3%)
Wetland was being used for aquaculture	1254 (± 375) (508 ha)	6.2% (± 1.9%)	8.7% (± 15.4%)		9.4% (± 15.0%)	22.6% (± 8.0%)	0.1% (± 0.1%)	0.2% (± 0.3%)	3.8% (± 5.8%)
Target population: wetland presumed to be target based on S&T categories	94,914 (± 5719)) (38,410 ha)	82.4% (± 2.1%)	98.1% (± 1.1)	99.8% (± 0.2%)	88.2% (± 2.9%)	41.6% (± 13.5)	19.5% (± 8.9%)	91.9% (± 2.5%)	92.0% (± 2.3%)
Sampled population: wetlands sampled as part of the NWCA, and, therefore, can be reported on	62,156 (± 5277) (25,154 ha)	65.5% (± 3.9%)	89.1% (± 4.0%)	50.3% (± 16.8%)	58.1% (± 5.5%)	12.5% (± 10.7%)	49.8% (± 35.3%)	66.5% (± 8.8%)	69.6% (± 6.8%)
Wetlands that are target, but cannot be assessed (sampled) for the following reasons:	32,757 (± 5277) (13,286 ha)	34.5% (± 3.9%)	10.9% (± 4.0%)	49.7% (± 16.8%)	41.9% (± 5.5%)	87.5% (± 10.7%)	50.2% (± 35.3%)	33.5% (± 8.8%)	30.4% (± 6.8%)
Landowner permission to access the wetland denied	23,462 (± 1873) (9495 ha)	24.7% (± 3.5%)	5.7% (± 3.3%)	7.6% (5.7%)	33.9% (± 5.0%)	41.6% (± 22.5%	36.05 (± 29.7%)	21.4% (± 7.4%)	22.9% (± 6.4%)
Too physically difficult to access	6041 (± 968) (2445 ha)	6.4% (± 2.0)	4.6% (± 2.0%)	40.3% (± 14.4%)	4.9% (± 1.9%)	23.5% (± 29.9%)	11.0% (± 13.4%)	5.8% (± 3.5%)	5.6% (± 3.3%)
Unsafe to access	407 (± 340) (165 ha)	0.4% (0.5%)	0.3% (± 0.3%)					2.4% (± 2.8%)	
AA area less than 0.5 ha	1577 (± 408) (638 ha)	1.7% (± 0.8%)	0.2% (± 0.3%)	0.1% (± 0.1%)	< 0.1% (<± 0.1%)	12.1% (± 12.7%)	0.1% (± 0.2%)	0.1% (± 0.1%)	0.5% (± 0.7%)
More than 10% of the AA unsampleable	541 (± 228) (219 ha)	0.6% (± 0.5%)		1.6% (± 2.8%)		8.2% (± 6.0%)	1.9% (2.8%)	3.0% (± 3.8%)	0.9% (± 0.8%)
AA crosses HGM classes	38 (± 28) (15 ha)	< 0.1% (± 0.1%)			0.1% (± 0.2%)	0.3% (± 0.5%)			
Unsafe or unable to sample due to impenetrable poison sumac, incised creek, overgrazed, etc.	685 (± 202) (277 ha)	0.7% (± 0.4)	0.2% (± 0.3%)		1.0% (± 0.8%)	1.6% (± 2.45)	1.0% (± 0.8%)	0.7% (± 0.8%)	0.6% (± 0.7%)
Too close to another NWCA sampling point so area sampled would overlap	6 (± 3) (3 ha)	< 0.1% (< 0.1%)				0.1% (± 0.2%)	0.2% (± 0.1%)		

See Table 1 for definitions of wetland types and acronyms. AA = assessment area, HGM = hydrogeomorphic class, MoE = margin of error

**Fig. 3:**
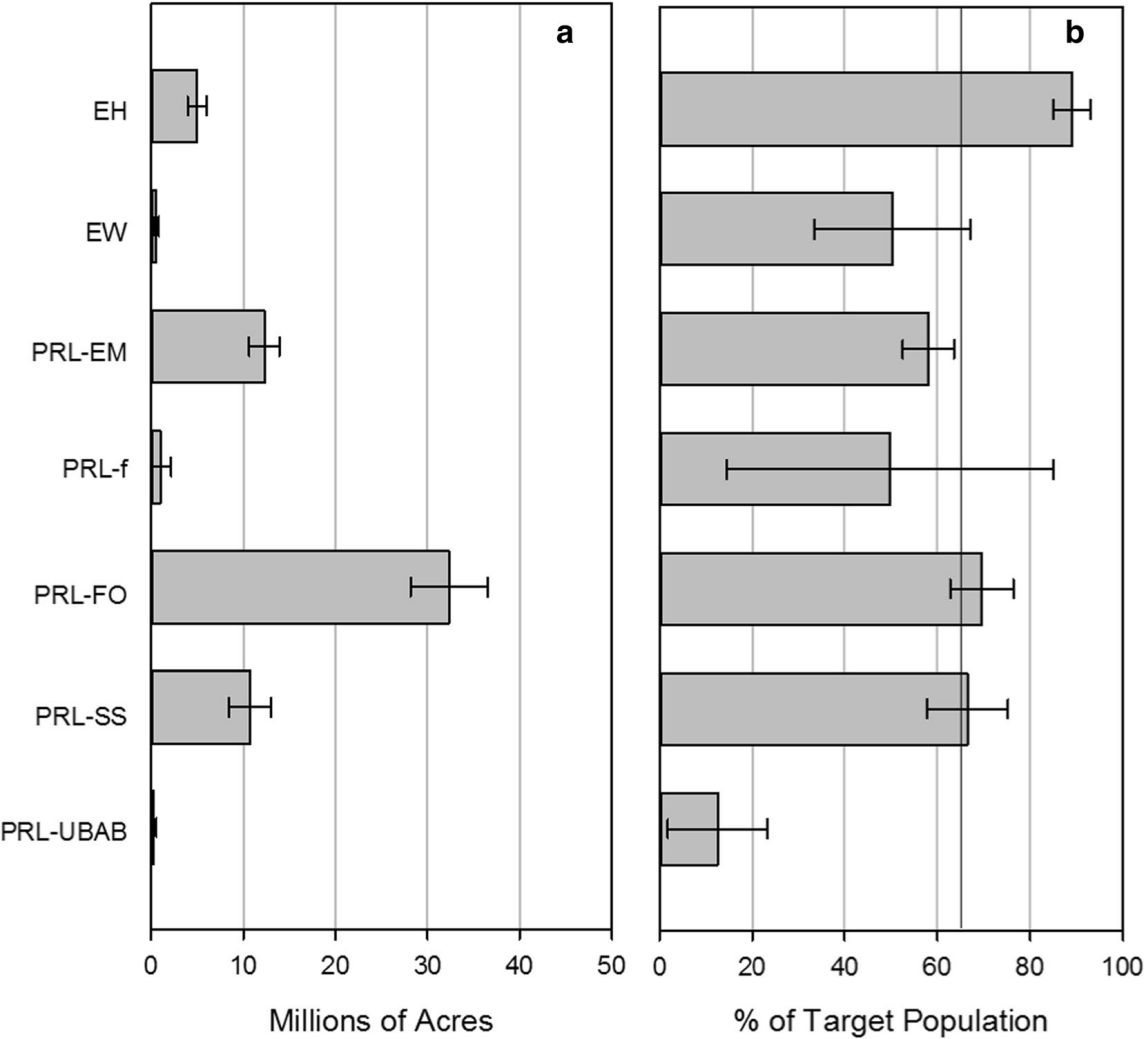
The area of the NWCA 2011 sampled population by wetland type (millions of acres) (**a**) and as a percent of the target population (**b**). See Table 1 for definitions of acronyms. The vertical line in panel **b** indicates the percent of the target population that could have been sampled if there were no issues with accessibility or in the field that prevented sampling a site. Error bars are 95% confidence intervals

Not all of the estimated target population acres could be sampled due to accessibility and field issues. Based on the sites that could be sampled, the sampled population for the NWCA is estimated to be 62.2 (± 5.28) million acres of wetland area (Table 4). In other words, the NWCA 2011 sampled population represented 65.5% (± 3.9%) of the estimated area of the target population (Table 4). The sampled population is estimated to be the highest percent of the target population for EH (89.1% ± 4.0%) and PRL_FO (69.6% ± 6.8%) (Fig. 3a, Table 4), while the sampled population is estimated to be the lowest percent of the target population for PRL-UBAB (12.5% ± 10.7%), PRL-f (49.8% ± 35.3%), EW (50.3% ± 16.8%), and PRL-EM (58.1% ± 5.5%) (Fig. 3b, Table 4). It is also important to note that the wetland areas of the sampled population (62.2 ± 5.28 million acres) as well as target population (94.9 ± 6.20 million acres) were less than that reported by the USFWS S&T (reported as 110 million acres in Dahl (2011)), in part because the NWCA wetland types are a subset of those reported by S&T.

**Fig. 4:**
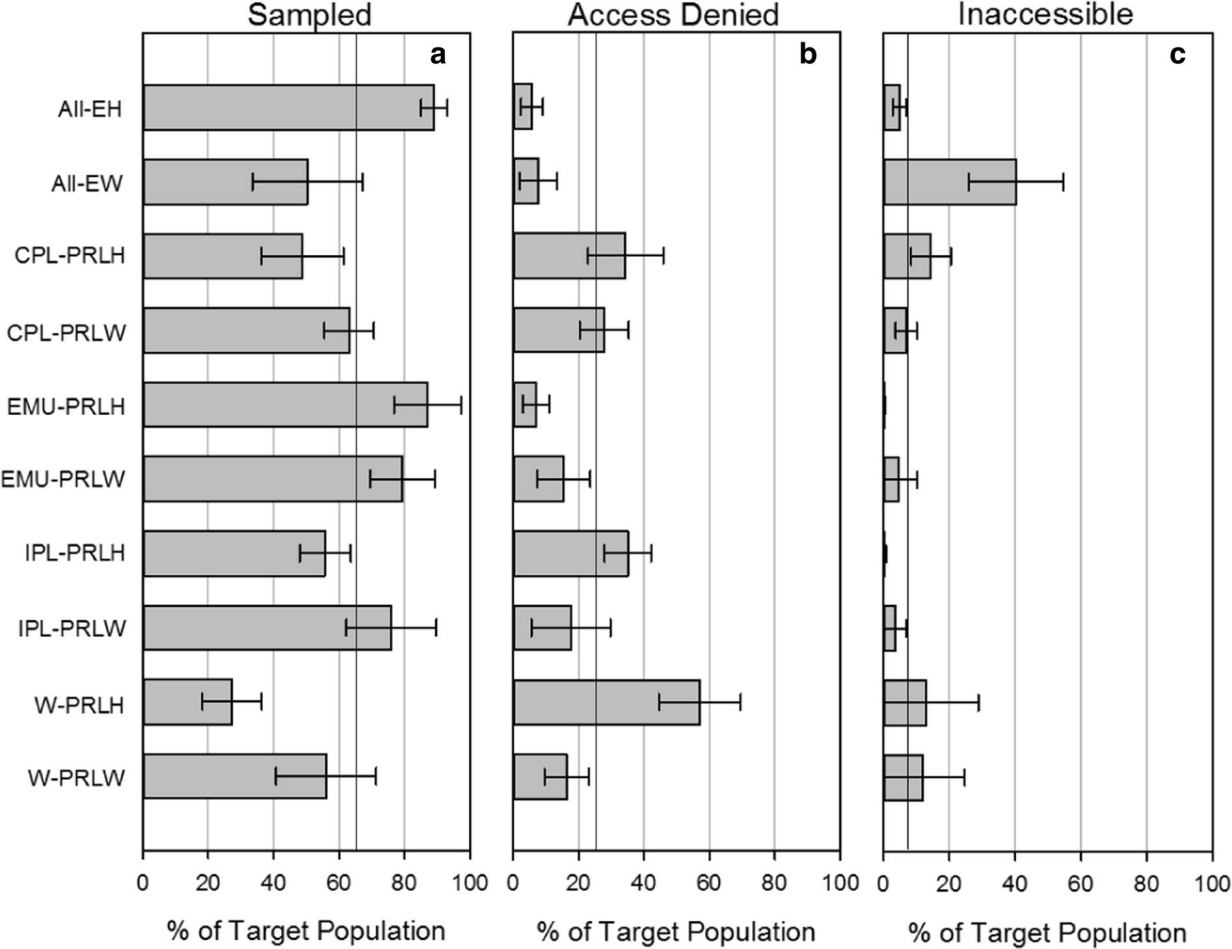
Percent of the target population that could be sampled (**a**) was denied access by landowner (**b**) and was physically inaccessible (**c**) by NWCA 2011 reporting unit. The vertical line in panel **a** at 65% is the national percent of the target population estimated to be the sampled population. Similarly, 25 and 7% vertical lines in panels **b** and **c** are the national percent of the target population that is estimated to have landowners deny access (**b**) or to be physically inaccessible due to effort (**c**). Regions, CPL, EMU, IPL, and W are as described in Fig. 1. See Fig. 1 and Table 2 for definitions of acronyms. Error bars are 95% confidence intervals

While landowner denial was the main reason overall (24.7% ± 3.5%) for the sampled population being smaller than the target population and physical inaccessibility was the second reason (6.8% ± 2.1%) (Table 4), the primary reason for not sampling a site varied by NWCA reporting unit (Fig. 4b, c). The highest landowner denial for access was for PRL-H in the West (57.1% ± 1.25%) followed by PRL-H in the Interior Plains (35.1% ± 7.1%), PRL-H in the Coastal Plains (34.4% ± 11.5%), and PRL-W in the Coastal Plains (27.9% ± 7.5%). The highest percent that was physically inaccessible by NWCA reporting unit is EW (40.3 ± 14.4%), PRL-H in Coastal Plains (14.4 ± 6.1%), PRL-H in West (13.0% ± 15.8%), and PRL-W in West (12.1% ± 12.

**Fig. 5:**
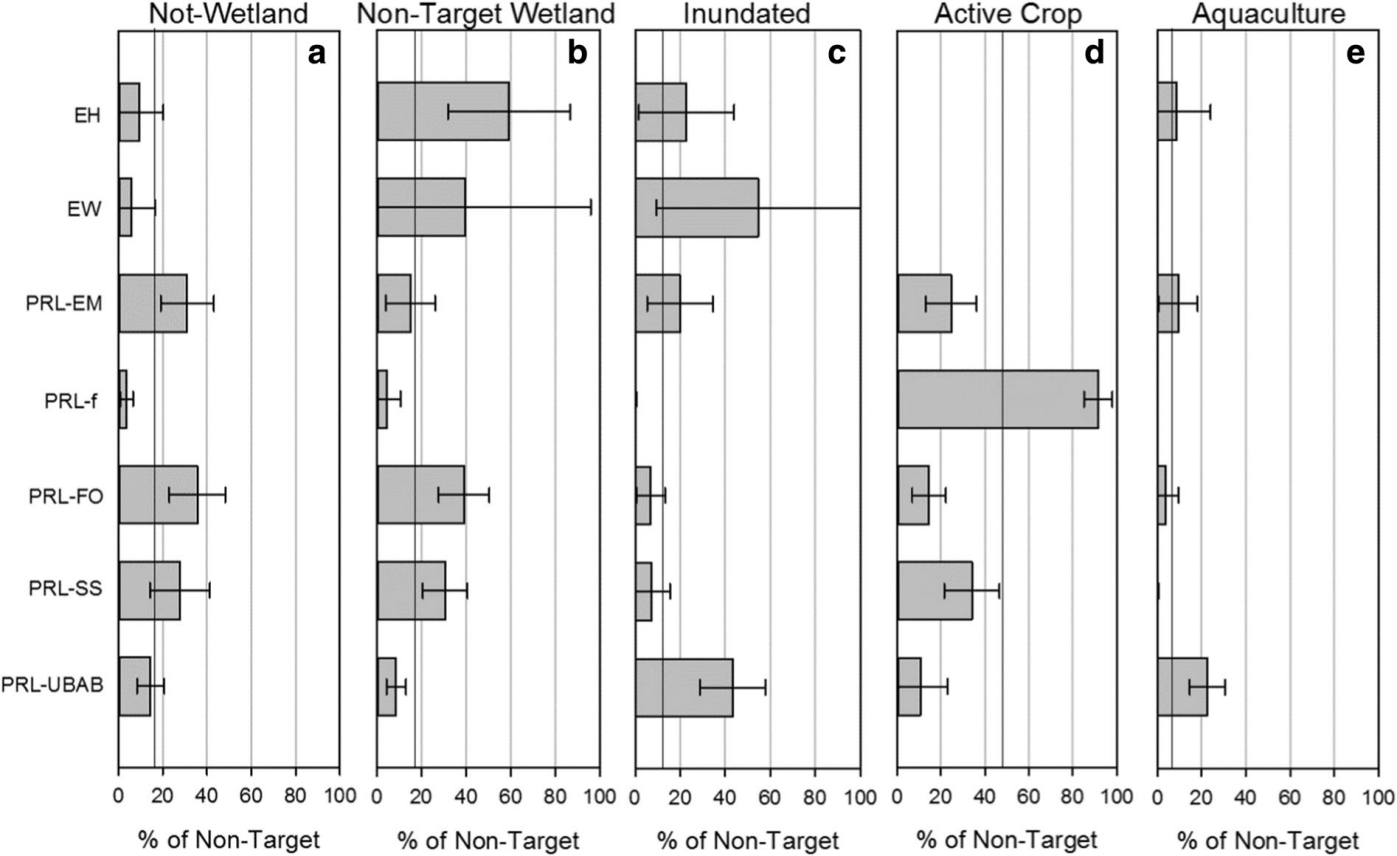
Reasons for sites being designated non-target by NWCA wetland type. See Table 1 for definitions of acronyms. The vertical lines in each panel indicate the value across all wetland types across the nation. Error bars are 95% confidence intervals

The site evaluations also provided information on how the sample frame wetland designations differed from those observed in the field. That is, why was a site non-target for the NWCA 2011? The primary reason for sites being non-target, in terms of percent of the target population (not area), was the wetland was being actively cropped (48.0% ± 6.3%) (Table 4). Over 90% of farmed wetlands were actively cropped, while between 11 and 34% of PRL-SS, PRL-EM, PRL-FO, and PRL-UBAB were actively cropped (Fig. 5d). Nationally, 17.5% ± 13.8% of the non-target wetlands category was not a wetland. This was highest for PRL-FO, PRL-EM, and PRL-SS where the percent ranged from 25 to 35% (Fig. 5a). Nationally, 15.7% ± 10.8% of the non-target wetlands were found to be a wetland, but the wetland type was not included in NWCA 2011 (Fig. 5b). This was highest for EW, EW, PRL-FO, and PRL-SS where the percent ranged from 30 to 59%. The fourth reason for being a non-target wetland was due to standing water on ≥ 90% of the potential site assessment area being too deep (“inundated”) to meet the NWCA wetland definition (12.6% ± 8.56%) (Fig. 5c). This was most common in EW, PRL-UBAB, EH, and PRL-EM wetland types, ranging from 20 to 55%. The least common reason for being a non-target wetland (6.2% ± 4.26%) was that the wetland was functioning as an aquaculture wetland with PRL-UBAB being the main wetland type (Fig. 5e).

## Discussion

The NWCA 2011 survey design was successful in enabling national and regional estimates of wetland condition to be made. Regional reporting consisted of ten reporting units based on a combinations of four ecoregions and four aggregated wetland types (palustrine, riverine, lacustrine herbaceous and woody, and estuarine herbaceous and woody). The design did link the NWCA with the USFWS S&T design. In addition to the connection between reporting on wetland area (S&T) and ecological condition (NWCA), the linkage with design was necessary because at the time the design was completed, no national digital coverage of wetlands existed for the contiguous USA. While the USFWS S&T design did provide a sample of wetland polygons that were delineated in a consistent manner and a consistent time period, it limited the options for the NWCA survey design. Most significant was that the S&T design focuses on national estimates only, resulting in most plots being located where wetlands are expected, while the NWCA design required not only national estimates but regional estimates as well. While the NWCA design did attempt to allocate sites in regions not well-covered by S&T, it was only partially successful, being most limited in the western half of the contiguous USA. Also, only having approximately 5000 4-mi^2^ plots available as the base of the NWCA design resulted in some clustering of sites within the plots. A sufficient number of wetland polygons for each wetland type were simply not available. This led to reducing the number of geographic regions and wetland types for reporting purposes.

For the NWCA 2016 survey design, we addressed the above issues by utilizing the underlying S&T survey design structure of 4-mi^2^ plots that cover the contiguous states. From S&T’s national coverage of 4-mi^2^ plots, we selected an additional 200 plots in each of the 48 states. Each of these plots was then populated with the wetland polygons from the US FWS national digital map data (http://www.fws.gov/wetlands/Data/Mapper.html). While the national digital map data is complete, it is compiled from the latest existing state-level wetland mapping effort. In many cases, the data are not current, are dominated by information from the 1970s and 1980s, and are mapped at different scales. While this is not ideal, it improves the wetland sample frame, particularly in the West, and likely includes more wetlands than are currently present due to the loss of wetland area since the 1970s. Wetlands constructed since the digital map data from a state was updated will be missing, leading to constructed wetlands being potentially under represented. The wetland classification used in the national digital map is the Cowardin wetland classification (Cowardin et al. 1979), and the S&T classification is based on the same classification but simplified. The more detailed classification used in the USFWS national digital map data enabled a more careful matching of wetland types to the NWCA wetland types. Overall, incorporation of the national digital map data improves the sample frame coverage of the NWCA target population and better meets the survey design requirement for geographic reporting units. While this resolves the issue of having more wetland sites in the west, it does so by giving up having current wetland polygon information. This results in the exclusion of newly constructed wetlands and possibly additional site evaluation due to wetlands disappearing, since the maps were constructed. Further integration of the USFWS S&T survey for wetland area and NWCA survey for wetland condition is an opportunity that could lead to an improvement in both programs.

One conclusion that can be made based on the number of sites evaluated is that if a similar design is used for the next NWCA survey, approximately two to three times the expected sample size would be required to result in 900 sampled wetland sites. Approximately one third of the sites evaluated would be determined to be non-target NWCA wetland types due to being located in an upland or deep water setting (i.e., not meeting definition of a wetland), being in a wetland type but not included in the NWCA wetland types, being in an actively cropped wetland, being in an aquaculture wetland, or being in an open water area of a wetland too deep (inundated > 1 m) to sample. The reason for being non-target would differ by NWCA wetland type. Given that a site would be evaluated as an NWCA wetland type, approximately 40% of the time the site would not be able to be sampled. The main reasons would be denial of access by landowner and the excessive physical effort necessary to reach the wetland site. The NWCA sample frame correctly identified the NWCA wetland type 82% of the time. Farmed wetlands (20%) and open water ponds and aquatic bed wetlands (42%) were the only two wetland types that were not well identified. In the former case, most were actively cropped wetlands, and in the latter, most had surface water too deep to meet the definition of a wetland. Given that wetland type within a state was ignored when selecting replacement sites, the impact was that not as many of these two wetland types were sampled as expected. It also resulted in additional costs due to the effort necessary to complete the site evaluations of the non-target wetland sites.

Nationally, 65% of the NWCA wetland-type target population was estimated to be sampleable, 25% was estimated non-sampleable due to landowner access denial, and 7% was estimated to be physically inaccessible. Wetlands that were physically inaccessible may be more likely to have less local anthropogenic stressors; consequently, they may more likely be in better condition than wetlands that are accessible. While this may be a potential source of bias in the percent of the target population that was in good condition, the impact will be minimal given the small percent (7%) that were inaccessible. Landowner access denial may have a greater impact due to the percent of access denial (25%) and due to private landowners being the source of the denials. PRL-W wetlands in Coastal Plains and Eastern Mountains and Upper Midwest had the most wetland acres with landowner denials (approximately 10 and 3 million acres, respectively). PRL-H wetlands in West, Interior Plains, and Coastal Plains regions were next (approximately 3 million acres each) with the West having the highest landowner denial rate (57%). Whether this results in any potential bias depends on whether wetlands associated with private landowners who deny access differ from wetlands associated with landowners who grant access. Note that Stevens Jr. and Jensen (2007) present methods that could be used to remove such bias.

The NWCA 2011 survey design was successful in enabling a national survey for wetland condition to be conducted and coordinated with the USFWS S&T survey of wetland extent. The NWCA 2016 survey design was modified to address sample frame issues resulting from the difference in S&T focusing only on national estimates and NWCA focusing on national and regional estimates. While 65% of the target population being sampled for NWCA 2011 is lower than for National Aquatic Resource Surveys of streams, lakes, and coastal waters (US EPA 2009, 2015, 2016), estimates of condition for the sampled population remain valid, although a potential bias in the estimates exists if the results are inappropriately assumed to apply to the target population of NWCA wetland types.

## References

[CR1] R Core Team. (2015). R: a language and environment for statistical computing. R Foundation for Statistical Computing, Vienna, Austria. http://www.R-project.org.

[CR2] Cowardin LM, Carter V, Golet FC, LaRoe ET (1979). Classification of wetlands and deepwater habitats of the United States.

[CR3] Dahl TE (1990). Wetlands: losses in the United States, 1780’s to 1980’s.

[CR4] Dahl TE (2000). Status and trends of wetlands in conterminous United States 1986 to 1997.

[CR5] Dahl TE (2006). Status and trends of wetlands in the conterminous United States 1998 to 2004.

[CR6] Dahl TE (2011). Status and trends of wetlands in the conterminous United States 2004 to 2009.

[CR7] Dahl TE, Bergeson MT (2009). Technical procedures for conducting status and trends of the nation’s wetlands..

[CR8] Dahl TE (1991). Status and trends of wetlands in the conterminous United States, mid-1970’s to Mid-1980’s.

[CR9] Diaz-Ramos S, Stevens DL, Olsen AR (1996). EMAP statistics methods manual.

[CR10] Ernst TL, Leibowitz NC, Roose D, Stehman S, Urquhart NS (1995). Evaluation of US environmental monitoring and assessment program’s (EMAP)—wetlands sampling design and classification. Environmental Management.

[CR11] Fennessy MS, Mack JJ, Deimeke E, Sullivan MT, Bishop J, Cohen M (2007). Assessment of wetlands in the Cuyahoga River watershed of northeast Ohio.

[CR12] Frayer, W. E., et al. (1983). *Status and trends of wetlands and deepwater habitats in the conterminous United States, 1950s to 1970s, Washington, DC* (p. 31). U.S. Department of the Interior, Fish and Wildlife Service.

[CR13] Genet, J. A. (2012). *Status and trends in wetlands in Minnesota: depressional wetland quality baseline*. Saint Paul, MN: Minnesota Pollution Control Agency.

[CR14] Genet JA, Olsen AR (2008). Assessing depressional wetland quantity and quality using a probabilistic sampling design in the Redwood River watershed, Minnesota, USA. Wetlands.

[CR15] Jacobs AD, Kentula ME, Herlihy AT (2010). Developing an index of wetland condition from ecological data: an example using HGM functional variables from the Nanticoke watershed, USA. Ecological Indicators.

[CR16] Kincaid, T. M. and Olsen, A. R. (2015). spsurvey: spatial survey design and analysis. R package version 3.1. URL: https://cran.fhcrc.org/. 10.18637/jss.v105.i03 PMC9926341 36798141

[CR17] Kloiber SM (2010). Status and trends of wetlands in Minnesota: wetland quantity baseline.

[CR18] Leibowitz NC (1991). Research plan for monitoring wetland ecosystems.

[CR19] Leibowitz NC (1993). Evaluation of EMAP—wetlands sampling design using national wetlands inventory data.

[CR20] Lesser VM (2001). Applying survey research methods to account for denied access to research sites on private property. Wetlands.

[CR21] Minnesota Pollution Control Agency (2015). Status and trends of wetlands in Minnesota: vegetation quality baseline.

[CR22] Nestlerode J, Engle V, Bourgeois P, Heitmuller P, Macauley J, Allen Y (2009). An integrated approach to assess broad-scale condition of coastal wetlands—the Gulf of Mexico coastal wetlands pilot survey. Environmental Monitoring and Assessment.

[CR23] Nestlerode JA, Hansen VD, Teague A, Harwell MC (2014). Application of a three-tier framework to assess ecological condition of Gulf of Mexico coastal wetlands. Environmental Monitoring and Assessment.

[CR24] Olsen AR, Peck DV (2008). Monitoring design and extent estimates for national. Wadeable Stream Assessment Journal of North American Benthological Society.

[CR25] Olsen AR, Kincaid TM, Payton Q, Gitzen RA, Millspaugh JJ, Cooper AB, Licht DS (2012). Spatially balanced survey designs for natural resources. Design and analysis of long-term ecological monitoring studies.

[CR26] Omernik JM (1987). Ecoregions of the conterminous United States. Annals of Association of American Geographers.

[CR27] Omernik JM, Griffith GE (2014). Ecoregions of the conterminous United States: evolution of a hierarchical spatial framework. Environmental Management.

[CR28] Peck DV, Olsen AR, Weber MH, Paulsen SG, Peterson C, Holdsworth SM (2013). Survey design and extent estimates for the National Lakes Assessment. Freshwater Sciences.

[CR29] Stevens DL, Jensen SF (2007). Survey design, execution, and analysis for wetland assessment. Wetlands.

[CR30] Stevens DL, Olsen AR (2003). Variance estimation for spatially balanced samples of environmental resources. Environmetrics.

[CR31] Stevens DL, Olsen AR (2004). Spatially-balanced sampling of natural resources. Journal of American Statistical Association.

[CR32] Turner RE, Swenson EM, Summers JK (1995). Coastal wetlands indicator study: EMAP-Estuaries Louisianian Province—1991.

[CR33] US EPA (2009). National lakes assessment: a collaborative survey of the nation’s lakes.

[CR34] US EPA (2011). National Wetland Condition Assessment 2001: field operations manual.

[CR35] US EPA (2015). National Coastal Condition Assessment 2010.

[CR36] US EPA (2016). National Rivers and Streams Assessment 2008–2009: a collaborative survey.

[CR37] Wardrop DH, Kentula ME, Jensen SF, Stevens DL, Hychka KC, Brooks R (2007). Assessment of wetlands in the upper Juniata watershed in Pennsylvania, USA, using the hydrogeomorphic approach. Wetlands.

[CR38] Wardrop DH, Kentula ME, Stevens DL, Jensen SF, Brooks RP (2007). Assessment of wetland condition: an example from the Upper Juniata Watershed in Pennsylvania, USA. Wetlands.

[CR39] Wardrop DH, Kentula ME, Brooks RP, Fennessy MS, Chamberlain S, Havens K, Brooks RP, Wardrop DH (2013). Monitoring and assessment of wetlands: concepts, case studies, and lessons learned. Mid-Atlantic freshwater wetlands: advances in wetlands science, management, policy, and practice.

[CR40] Whigham DF, Jacobs AD, Weller DE, Jordan TE, Kentula ME, Jensen SF, Stevens DL (2007). Combining HGM and EMAP procedures to assess wetlands at the watershed scale -status of flats and non-tidal riverine wetland in the Nanticoke River watershed, Delaware and Maryland (USA). Wetlands.

